# Reduction of SARS-CoV-2 viral load in exhaled air by antiseptic chewing gum: a pilot trial

**DOI:** 10.1007/s15010-022-01944-2

**Published:** 2022-10-19

**Authors:** F. Pfab, B. Buelow-Johansen, D. Alber, M. Kriner, O. Kornmann, M. Stuermer

**Affiliations:** 1 Department of Dermatology and Allergy, Technical University of Munich, Munich, Germany; 2 IKF Clinical Research Centre Respiratory Medicine, Frankfurt, Germany; 3 Infektiologikum Frankfurt, Frankfurt, Germany; 4 Statistical Consultant, Munich, Germany; 5 IMD Labor, Frankfurt, Germany

**Keywords:** Cinnamon, Peppermint, Lemon, Ginger, Ginseng, Pandemic

## Abstract

**Purpose:**

The dominant route of transmission of SARS-CoV-2 is airborne, through respiratory transmission by aerosols or droplets which can be measured by viral load in exhaled air. Several natural substances have shown antiviral activity. The aim of this pilot study was to investigate the effect of a chewing gum containing natural antiseptic ingredients (cinnamon-, peppermint- and lemon-oil, quercetin, spermidine, ginger and ginseng) on viral load in exhalative air in patients infected with SARS-CoV-2.

**Methods:**

Nine patients infected with SARS-CoV-2 were enrolled and exhaled forcefully into a special mouthpiece at different time points before and after chewing the antiseptic gum. The mouthpiece contained a filter paper serving for extraction of coronaviruses following real-time PCR to quantify the viral load.

**Results and conclusion:**

Cycle threshold (Ct) values of all patients increased after chewing the gum. The mean difference between the Ct values at baseline (before chewing the antiseptic gum) and time point 30 min (15 min after chewing) was 3.8 ± 2.6; (93% viral load reduction; *p* = 0.002). Time point 15 min (2.7 ± 1.7 (83% viral load reduction; *p* = 0.003)), 60 min (3.0 ± 3.4 (88% viral load reduction; *p* = 0.028)), 90 min (3.7 ± 1.8 (92% viral load reduction; *p* = 0.004)) and 120 min (3.0 ± 3.7 (91% viral load reduction; *p* = 0.05)) showed similar results. The antiseptic chewing gum demonstrated a significant potential to reduce SARS-CoV-2 viral load in exhalative air and, in this way, reduce further spread and infection risk. Larger placebo-controlled clinical trials are required to confirm these findings further.

## Introduction

The coronavirus disease 2019 (COVID-19) caused by severe acute respiratory syndrome coronavirus 2 (SARS-CoV-2) has turned in the current high impact pandemic. The dominant route of transmission of SARS-CoV-2 is airborne through respiratory transmission by aerosols or droplets [1].

The oral cavity is an important site for a SARS-CoV-2 infection, and saliva a potential route of SARS-CoV-2 transmission [2]. Oral viral load has been associated with severity of COVID-19 [3]. The current hygiene measures aim at reducing the spread of viral load from leaving the oral cavity. Antiseptic mouth rinses have shown promise to reduce viral load in the oral cavity and act as a preventive measure [4, 5]. However, they are rather complicated to use in different setups and populations. Depending on the situation, chewing gums or lozenges containing antiseptic volatile substances are easier to use than mouth rinses. Furthermore, they can be kept in the oral cavity for plenty of time and release their ingredients slowly and continuously. There are several antiseptic natural substances that have been shown to exhibit antiviral effects and seemed promising as potential ingredients for developing an antiviral chewing gum or lozenge [6].

The aim of this pilot trial was to investigate the effect of an antiviral chewing gum on viral load in exhalative air in patients infected with SARS-CoV-2.

## Methods

The viral load in exhaled air was examined in patients infected with SARS-CoV-2 via PCR analysis before and after chewing an antiseptic chewing gum at different time points.

Cycle Threshold (Ct) values were generated by real-time PCR performed on the Cobas 6800 (Roche) detecting the ORF1 a/b and the E-gene of SARS-CoV-2 using the CE-certified test by Roche.

Before that, swabs had been sent to the Konsiliarlabor für Coronaviren (laboratory for corona virus) at the Institute of Virology, Charité-Universitätsmedizin Berlin, Germany for analyzing the Variants of Concern (VoC).

### Subjects

Inclusion criteria: Volunteer adult (age over 18 years) patients suffering from COVID-19 with a positive PCR test confirming infection and being able to easily sit in an isolation room for two hours.

### Study design

This prove of concept study was conducted at the IKF Clinical Research Center Respiratory Diseases, Frankfurt, Germany. PCR testing was done by Infektiologikum Frankfurt, Zentrum für fachübergreifende Infektionsmedizin (center for multidisciplinary infectious medicine). Approval of local ethics committee was granted; written consent was obtained, and the study was conducted in accordance with the declarations of Helsinki.

At each sample collection time point patients exhaled forcefully after a maximal inspiration through a mouthpiece containing a special filter paper. A predefined area of the filter paper was used for PCR analysis. The Ct value was used as marker for SARS-CoV-2 viral load. Ct is a theoretical parameter in PCR analysis. The Ct value shows the point in time at which a reliable measurement signal is present during the cyclic amplification: the lower the Ct value, the higher the amount of the target gene (corresponding nucleic acid) and thus the amount of virus particles. Conversely, a high Ct value indicates a low amount of nucleic acid and possibly a low virus titer.

Sample collection for the antiseptic chewing gum intervention occurred directly before (*t* = 0 min) and after a 15 min period (*t* = 15 min) of chewing, as well as 30 min (*t* = 30 min), 45 min (*t* = 45 min), 60 min (*t* = 60 min), 90 min (*t* = 90 min) and 120 min (*t* = 120 min) after the first sample collection.

### Antiseptic gum active ingredients

In our study, a commercially available chewing gum (Clevergum GmbH, Munich, Germany) containing essential oils (cinnamon, lemon, peppermint) and extracts (ginger, ginseng, quercetin and spermidine) was used.

### Statistical analysis

Main outcome parameter: Mean difference between the Ct values of time point 45 min (1/2 h after chewing) and baseline.

Further outcome parameters: Mean differences between each of the Ct values at the other time points after chewing (15 min, 30 min, 60 min, 75 min, 120 min) and baseline.

Differences between sample collection time points were evaluated using multiple paired samples *t* tests.

The global significance level was set to 0.05 and all statistical tests were two-sided. If not mentioned otherwise, mean values ± standard deviation is given.

## Results

Nine patients infected with SARS-CoV-2 between 18 and 64 years took part in this pilot trial. All patients were symptomatic for at least one day and a maximum of six days. Four patients had wild type, three alpha and two delta SARS-CoV-2 variants.

The Ct values before chewing the gum (baseline, time point 0) were 29.9 ± 2.5, and increased in all patients after chewing the gum.

**Fig. 1:**
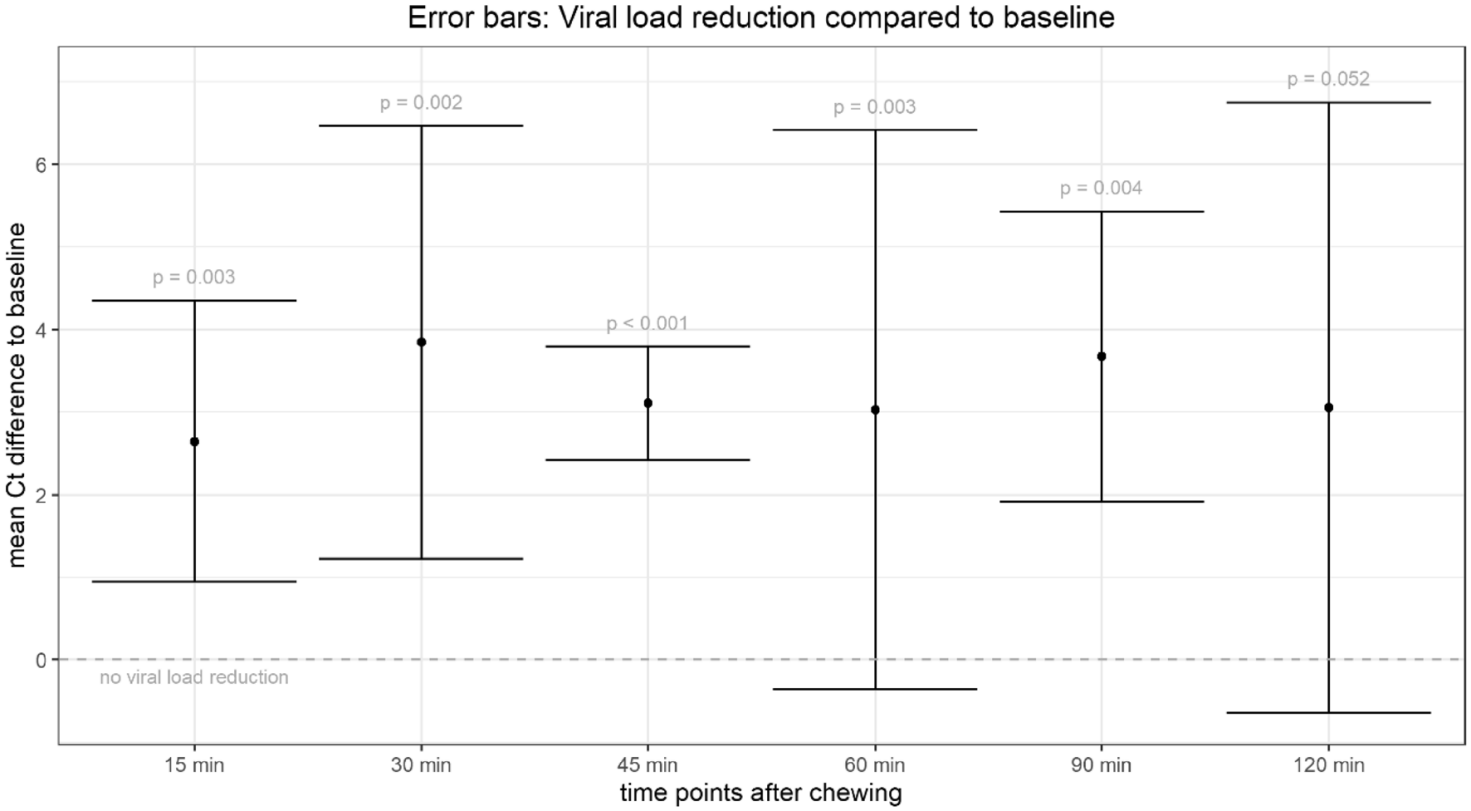
Differences of Ct values at different time points before chewing the antiseptic gum (baseline) and after having chewed the gum (all subsequent time points)

The mean difference between the Ct values at baseline (time point 0; before chewing the antiseptic gum) and time point 30 min (15 min after chewing) was 3.8 ± 2.6; (93% viral load reduction; *p* = 0.002). Figure 1 shows the viral load reduction as compared to baseline in terms of differences of Ct values to baseline as error bars (mean ± SD) for all time points and over all patients. All means lie over the zero line, which indicates viral load reduction.

**Fig. 2:**
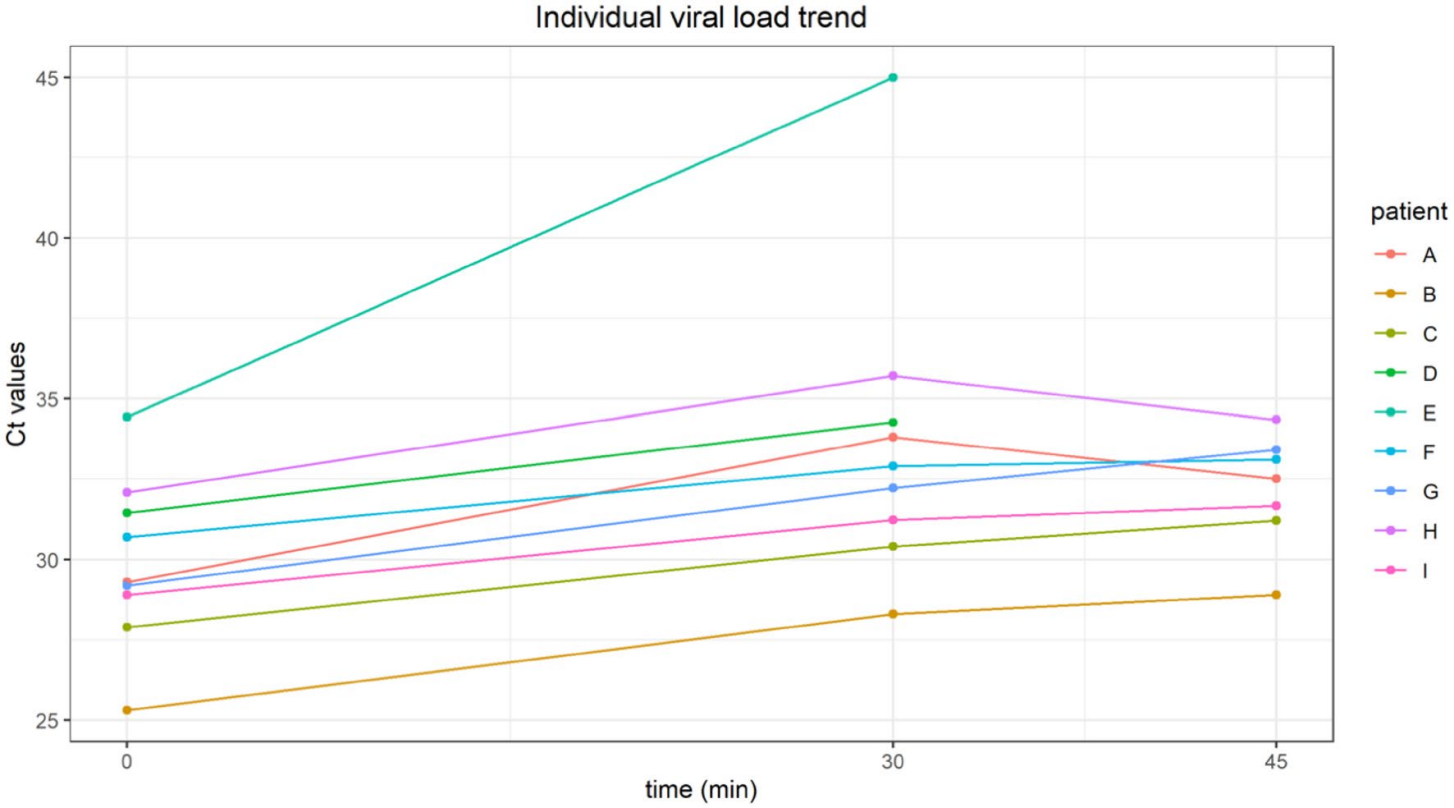
Individual viral load trend before (time = 0) and after chewing the antiseptic gum

When compared with baseline mean difference between the Ct values at time point 15 min (directly after chewing) was 2.7 ± 1.7 (83% viral load reduction; *p* = 0.003), at time point 45 min (1/2 h after chewing) 3.1 ± 0.7 (88% viral load reduction; *p* < 0.001), at time point 60 min 3.0 ± 3.4 (88% viral load reduction; *p* = 0.028), at time point 90 min 3.7 ± 1.8 (92% viral load reduction; *p* = 0.004) and at time point 120 min 3.0 ± 3.7 (91% viral load reduction; *p* = 0.05) (Fig. 1). Individual patient trends are shown in Fig. 2.

## Discussion

Viral load in exhaled air is supposedly a representative parameter for infection risk: Assessing the infectiousness of COVID-19 patients merely through pharyngeal swabs seems to not be accurate whereas exhaled breath was concluded to represent a more suitable matrix for evaluating the degree of infectiousness [7].

The active ingredients of the antiseptic gum have all shown to exhibit antiseptic effects. Cinnamon oil [8–12], extract [20–22] and ginger extract [9, 22] as well as spermidine [23] have shown strong antiseptic effects and peppermint oil [13, 14], citrus oil [12, 15–17], the flavonoid quercetin [18, 19] as well as saponin containing panax ginseng have shown antiviral effects in vitro. Lemon essential oils furthermore downregulate angiotensin-converting enzyme2 (ACE2), a SARS-CoV-2 spike receptor-binding domain in epithelial cells [24].

In this pilot study, patients that had chewed antiseptic chewing gums showed a significant reduction of viral load in exhaled air, indicated by increased Ct values as compared to baseline, for up to 120 min. Given a reduction of 50% of viral load per 1.0 increase of Ct value, viral load dropped between 84 and 93% on average within a 2 h period after intake of the chewing gum. These results point to a significant antiviral effect of the antiseptic chewing gum confirming the antiviral in vitro effect of its antiseptic ingredients.

The antiseptic gum used in this study is the first of such products on the market, so there is no comparable food product.

Mouth rinses developed for antiseptic mouth washing containing for example povidone iodide or chlorhexidine have shown promising effects on SARV-CoV-2 in vitro [25], however in vivo evidence is still limited and studies in COVID-19 patients examining exhaled air are lacking so far [4].

There are some limitations of this pilot study. We included a relatively small number of patients which showed expected individual trends of viral load reduction. This trial was designed as a “proof-of-principle”-study to investigate whether chewing an antiseptic gum can significantly impact reduction of viral load in exhaled air at all. It is further to mention that only wild type, alpha and delta variants were included. This is due to the fact, that the pilot trial was performed when no omicron variants were yet present in Germany. There may possibly be differences in the anatomic replication region for the different SARS-COV-2 variants. However, we expect an equal distribution of antiseptic gum ingredients in all relevant potential replication sites due to the continuous chewing process.

Another interesting question to address is whether chewing a gum by itself may—independent from the gum’s ingredients—increase saliva production and dilute or decrease viral load. We report a nearly tenfold reduction of viral load in exhaled air and do not consider increased saliva flow in the oral cavity solely or largely responsible for such a result.

In addition, an increased saliva flow in the oral cavity by chewing any type of gum will most probably not influence viral load measured in exhaled air to such an extend measured as in our study.

Despite that, we believe that it is necessary to rule out dilution or other effects by including a control group in future studies. In order to take into account the limitations of this pilot trial we have just started a larger placebo controlled trial including patients suffering from infection with omicron variants (trial number 2022-2870-evBO).

## Conclusion

The antiseptic chewing gum showed promise to reduce SARS-CoV-2 viral load in exhalative air and, by doing so, reduce infection risk. Larger placebo-controlled clinical trials will need to confirm these findings further.

## Data Availability

Study carried out at IKF Frankfurt. All data and material can be accessible on the website.
